# Cold Storage Extends Larval Release Windows of *Archanara neurica* and *Lenisa geminipuncta* (*Noctuidae*), Biological Control Agents for *Phragmites australis australis*

**DOI:** 10.3390/insects17020194

**Published:** 2026-02-12

**Authors:** Michael J. McTavish, Ian M. Jones, Carla Timm, Sandy M. Smith, Robert S. Bourchier

**Affiliations:** 1 Rare Charitable Research Reserve, 1679 Blair Road, Cambridge, ON N3H 4R8, Canada; mike.mctavish@raresites.org; 2 Institute of Forestry and Conservation, John H. Daniels Faculty of Architecture, Landscape and Design, University of Toronto, 33 Willcocks Street, Toronto, ON M5S 3B3, Canada

**Keywords:** biocontrol, egg development, hatch timing, insect rearing, phenology

## Abstract

Two biological control agents, *Archanara neurica* (Hübner) and *Lenisa geminipuncta* (Haworth) (Lepidoptera: Noctuidae), are being released in Canada for the control of invasive common reed, *Phragmites australis australis* (Cav.) Trin. Ex Steud (hereafter *Phragmites*). The release of larvae implanted in cut *Phragmites* stems is the most reliable way to establish agents at new sites, but the number of larvae that can be used for releases is limited by the short period of time over which egg hatch occurs. We conducted a cold storage experiment to assess whether the timing of egg hatch can be manipulated without affecting hatch success. Additionally, we conducted visual assessments of developing eggs to determine whether hatch timing can be predicted based on early signs of development. Eggs hatched indoors had lower hatch rates than eggs hatched in outdoor conditions. For *A. neurica* and *L. geminipuncta*, eggs could be held in cold storage for 11 and 8 weeks, respectively, without affecting hatch rates. Eggs of both species began hatching 4–7 days after the appearance of visible signs of larval development. Manipulating the timing of hatch in *A. neurica* and *L. geminipuncta* will increase the number of larval releases that can be conducted during the spring and allow the timing of releases to be optimized.

## Simple Summary

Two biological control agents, *Archanara neurica* and *Lenisa geminipuncta*, are being released in Canada for the control of invasive common reed, *Phragmites australis australis* (hereafter *Phragmites*). The release of larvae implanted in cut *Phragmites* stems is the most reliable way to establish agents at new sites, but the number of larvae that can be used for releases is limited by the short period of time over which egg hatch occurs. We conducted a cold storage experiment to assess whether the timing of egg hatch can be manipulated without affecting hatch success. Additionally, we conducted visual assessments of developing eggs to determine whether hatch timing can be predicted based on early signs of development. Eggs hatched indoors had lower hatch rates than eggs hatched in outdoor conditions. For *A. neurica* and *L. geminipuncta*, eggs could be held in cold storage for 11 and 8 weeks, respectively, without affecting hatch rates. Eggs of both species began hatching 4–7 days after the appearance of visible signs of larval development. Manipulating the timing of hatch in *A. neurica* and *L. geminipuncta* will increase the number of larval releases that can be conducted during the spring and allow the timing of releases to be optimized.

## 1. Introduction

Species introductions, intentional or otherwise, are aided by increased propagule pressure [1]. As such, a major challenge of implementing successful classical biological control programs is maximizing the number and size of agent releases [2]. Two biological control agents, *Archanara neurica* (Hübner) and *Lenisa geminipuncta* (Haworth) (Lepidoptera: Noctuidae), have been released for the control of *Phragmites australis australis* (Cav.) Trin. Ex Steud. (hereafter *Phragmites*) in Canada. Currently, the most effective release method for these agents uses the implantation of freshly hatched larvae into cut *Phragmites* stems [3]. This method is labor-intensive and has been limited to a short and unpredictable time period, when the eggs of laboratory-reared populations hatch in the spring [3]. We sought to improve our ability to predict and manipulate agent hatch timing, such that the released window and the number of larvae available to be used for releases could be increased.

Introduced *Phragmites* has been described as Canada’s worst weed [4]. Arriving in North America from Eurasia in the late 19th century [5], the weed is now highly invasive in the Atlantic, Pacific, and Gulf Coastal regions [6], with significant potential for further range expansion [7]. Introduced *Phragmites* is a perennial grass that forms dense stands in wetland habitats [8]. The weed reduces once diverse wetland flora to little more than a monoculture [9], with cascading impacts on native fauna, including birds [10], fish [11], and turtles [12]. The native North American subspecies of common reed (*Phragmites australis americanus* Saltonstall, P.M. Peterson & Soreng) is under intense pressure from its invasive counterpart, both as a result of competition [5,13] and the threat of hybridization [14], cases of which have been confirmed in the USA [15]. In addition to its devastating ecological effects, invasive *Phragmites* is a major threat to infrastructure, leading to high economic costs [16]. The weed is common on roadsides, where its height can block sightlines, and in drainage ditches and culverts, where its dense stems and robust rhizomes impede the natural flow of water [17].

Chemical control of invasive *Phragmites* can be effective, but is limited by site accessibility, restrictions on chemical use, and the scale of the infestation [18,19]. Mechanical control methods such as spading or cut-to-drown can be effective for managing small populations, but these populations remain vulnerable to reinvasion from surrounding areas [18,20,21]. Classical biological control represents a promising tool for the management of invasive *Phragmites*. Biological control can complement existing *Phragmites* management tools while offering scalability, sustainability, and reduced impacts on non-target species [22].

The biological control program for introduced *Phragmites* began in the late 1990s, with assessments of the weed as an appropriate target and a search for candidate agents [23,24,25]. The two moths *A. neurica* and *L. geminipuncta* were identified by CABI in Delémont, Switzerland. The agents share similar life histories and univoltine life cycles. Eggs hatch in the spring, and larvae feed internally on *Phragmites* stems [26]. Over the course of their development, individual moths will feed inside three to four *Phragmites* stems, either killing them or stunting their growth and reproductive development [27]. Adult moths emerge in midsummer and lay eggs under the leaf sheaths of standing *Phragmites* stems [28,29]. Eggs are the overwintering life stage and remain under the leaf sheaths until the following spring. After rigorous host-range testing [30,31], a petition to release both agents in Canada and the USA was submitted in 2018 [32]. The agents were permitted for release by the Canadian Food Inspection Agency in Canada in 2019. In the USA, the agents have been recommended for release by the USDA-APHIS Technical Advisory Group and are still under regulatory review.

Since 2019, release methods have been developed for both eggs and larvae of *A. neurica* and *L. geminipuncta*, with larval releases proving the most effective [3]. To conduct larval releases, newly hatched larvae are placed inside cut *Phragmites* stems, and these inoculated stems are mounted in florist foam blocks (usually in groups of 40) before being placed into release sites [3]. The process is labor-intensive, and the number of releases that can be conducted is further limited by the short period of time over which egg hatch occurs in laboratory-reared agent populations. We conducted a cold storage experiment with eggs to assess whether the timing of *A. neurica* and *L. geminipuncta* egg hatch can be manipulated and extended without affecting egg survival. The ability to delay egg hatch would enable biological control practitioners to conduct larval releases over a longer period of time and manipulate the timing of releases to better coincide with *Phragmites* emergence in the field. Additionally, we conducted visual assessments of developing agent eggs to determine whether hatch timing can be predicted based on early signs of development. The ability to predict waves of agent hatch would promote more efficient use of practitioner time and resources and, ultimately, increase the number of larvae that can be released.

## 2. Materials and Methods

### 2.1. Insect Production

Eggs of *Archanara neurica* and *Lenisa geminipuncta* were collected from a mix of sources, including overseas rearing conducted at CABI, Delémont, Switzerland (43% of total, *A. neurica*; 51% of total, *L. geminipuncta*); laboratory-based oviposition trials at the University of Toronto (35% of *A. neurica*, 33% of *L. geminipuncta*); dedicated laboratory-based rearing in oviposition cages at the University of Toronto (21% of *A. neurica*); and field collections of eggs from Ontario release sites (1% of *A. neurica*, 16% of *L. geminipuncta*). Eggs of each species were mixed and homogenized across sources prior to use in this experiment. All eggs were then stored in Petri dishes packed in insulated polystyrene boxes in outdoor sheds starting in September 2023 in Ontario, Canada, prior to use in 2024.

### 2.2. Assessing the Effects of Spring Cold Storage on the Eggs of A. neurica and L. geminipuncta

We conducted an experiment to assess the feasibility of using cold storage to manipulate the hatch timing of the biocontrol agents *A. neurica* and *L. geminipuncta*. Eggs of both species were allocated to seven storage treatments on 15 March 2024: (1) a baseline treatment of eggs that remained in outdoor storage; (2) an early hatch treatment in which eggs were brought into the laboratory to determine if eggs could be hatched earlier than normal; and (3–7) five cold storage (c. 5 °C) treatments lasting 7, 8, 9, 10, and 11 weeks to see if egg hatch could be delayed.

Outdoor baseline eggs were left in the same outdoor shed in Petri dishes (n = 5 dishes of 30 eggs per species). The indoor early hatch eggs were brought into the laboratory on 15 March 2024 and placed in Petri dishes on a laboratory bench (n = 3 dishes of 20 eggs per species; this was a lower number of replicates and eggs per dish to minimize the number of “wasted” larvae that hatched too early in the season to be used for field releases). The cold storage eggs were randomly allocated to Petri dishes (n = 10 dishes of 30 eggs per species per cold storage duration) and placed in a walk-in refrigerator (4.6 °C ± 1.5 °C, mean ± standard deviation). After 7–11 weeks of cold storage, eggs from egg cold storage treatment were either brought indoors to laboratory conditions (~25 °C, n = 5 dishes of 30 eggs per species per cold storage duration) or transferred to the outdoor shed (n = 5 dishes of 30 eggs per species per cold storage duration) to evaluate the effect of location/temperature on overall hatch.

Eggs were visually monitored every day for hatch. Once no additional hatch was observed, we calculated the overall hatch (% of initial eggs in each Petri dish that hatched) and the time to first hatch (number of days to first hatch after an egg group was transferred indoors [early indoor warmup treatment] or moved out of cold storage until the first egg hatched [cold storage treatments]).

A temperature recorder (LogTag, Lafayette, NJ, USA) was placed in each location to record hourly temperature throughout the experiment. Hourly temperature data were used to generate summary statistics characterizing the outdoor, indoor, and cold storage locations (Table 1). Hourly integral cumulative degree days were also modeled at hypothetical base development temperatures of 5 °C and 0 °C for each storage treatment to better characterize the storage conditions. The base temperature of 0 °C provided a general above-freezing heat-sum index for seasonal warming. The base temperature of 5 °C is a standardized reporting threshold to aid comparability across studies and approximates cool-season developmental thresholds in many temperate systems. The comparison of 0 °C and 5 °C thresholds allowed us to test the sensitivity of our results to different base temperatures in the absence of known lower development threshold temperatures for *A. neurica* and *L. geminipuncta* [33]. For each cold storage treatment, degree days were calculated from the start of the cold storage experiment on 15 March 2024 until the average date of first emergence for combined *A. neurica* and *L. geminipuncta* in outdoor conditions. The combined average dates of the first hatch for *A. neurica* and *L. geminipuncta* for the cold storage treatment were 5 May for the outdoor baseline, 27 March for the early hatch treatment, 15 May for 7 weeks cold, 17 May for 8 weeks cold, 22 May for 9 weeks cold, 28 May for 10 weeks cold, and 5 June for 11 weeks cold.

### 2.3. Monitoring Visual Signs of Egg Development to Predict Hatch Timing

**Figure 1:**
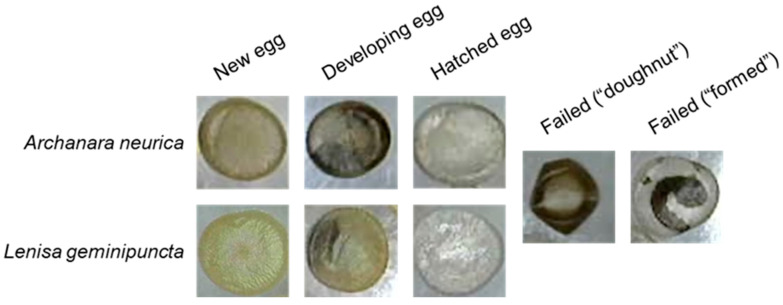
Egg classifications for *Archanara neurica* and *Lenisa geminipuncta* photographic monitoring.

To assess how visual evidence of egg development might be used to predict hatch timing, one dish of eggs in each of the outdoor and indoor treatments for each species was designated for photographic monitoring. Dishes were photographed every 2–3 days to visually assess developmental status. Photographs were taken using a Dino-Lite AM3111 0.3 MP Digital Microscope (Dunwell Tech, Inc. Dino-Lite, Torrance, CA, USA). The replicates from the outdoor treatment were brought indoors long enough to take photographs before being moved back outside. Photographs were visually reviewed, and eggs were classified into different developmental categories. All eggs began as “new” eggs perceived to be healthy and viable with no visible signs of development. Eggs that had not yet hatched but contained discernible internal embryonation or other structures with green to brown pigmentation on the egg surface were classified as “developing eggs”. Clear, empty eggs were classified as “hatched”. At the end of the observation period and after no further hatch was observed, eggs could be sorted into one of two common failed hatch conditions: failed eggs either took on a “doughnut”-like appearance with a ring of pigmentation and puckering around the edge or appeared as a mostly clear egg with a dark, desiccated, partially “formed” larva inside (Figure 1).

### 2.4. Statistical Analyses

The effects of species (*A. neurica*, *L. geminipuncta*), storage conditions (outdoor baseline, indoor early hatch, 7 weeks cold, 8 weeks cold, 9 weeks cold, 10 weeks cold, 11 weeks cold), and hatch location (indoors or outdoors) on final hatch were initially assessed using a general linear model (final hatch~species × storage conditions × hatch location). Because eggs in the indoor hatch location treatment had particularly low hatch rates, subsequent models included only replicates from the outdoor hatch location treatment. The effects of species and storage conditions on time to final hatch and time to first hatch were then assessed using general linear models (response~species × storage conditions). The outdoor baseline treatment was omitted from the analysis of time to first hatch since there was no discrete start time to measure against for eggs that remained in outdoor storage throughout the experiment. Assumptions of residual normality and equal variance between treatment groups were visually assessed by examining normal quantile (QQ) plots of residuals and boxplots of response variables by treatments, respectively. Means throughout are presented ± standard deviation (SD) as a measure of variation. Statistically significant general linear model results are presented with partial omega squared (ω_p_^2^) as an effect size, describing the proportion of variance explained by a model term while excluding the variance contributed by other terms.

For the photographic monitoring, visual records of egg development were used to produce descriptive summaries of the percentages of eggs that hatched or failed, the number of days between the first signs of development and first hatch, and the total duration of the hatch period (i.e., number of days from first to last hatch). Because of the time-intensive nature of the photographic monitoring, only one dish from each of the outdoor and indoor treatments for *A. neurica* and *L. geminipuncta* (four dishes in total) was processed, and no formal statistical inferences could be drawn.

Statistical analyses were conducted in R Studio version 2023.12.1 [34] at α = 0.05. Graphics were produced in R Studio, Microsoft Excel, and Microsoft PowerPoint.

## 3. Results

### 3.1. Assessing the Effects of Spring Cold Storage on the Eggs of A. neurica and L. geminipuncta

Temperature conditions varied between the outdoor, cold storage, and indoor locations in the egg cold storage experiment (Table 1). Overall, the outdoor courtyard was the most variable and experienced the broadest range of temperatures, including the lowest temperature (−4.3 °C). The cold storage refrigerator experienced brief spikes in temperature associated primarily with the door of the walk-in refrigerator being opened but on average maintained a temperature of 4.6 °C throughout the experiment. Degree days accumulated most rapidly in the indoor environment, followed by the outdoor courtyard (Table 2). Assuming a base temperature of 5 °C, degree day accumulation in cold storage was low but not zero because of fluctuations in cold room temperature. Using a development threshold of 5 °C, degree day accumulation was highest under the outdoor and early hatch conditions and lower for eggs held in cold storage (Table 2). Using a lower threshold of 0 °C, cumulative degree day accumulation was more consistent between all groups, except for the early hatch group, which had lower accumulation (Table 2).

When all eggs were analyzed together (indoor and outdoor treatments for both *A. neurica* and *L. geminipuncta*), egg hatch percentage was significantly affected by hatch location (indoors versus outdoors) (F_6,52_ = 336.172, *p* < 0.001). Eggs held indoors after cold storage (24.3 ± 2.1) hatched at a significantly lower rate than eggs held outdoors (66.6 ± 2.6). Eggs in the early hatch treatment (brought indoors on 15 March) hatched 38 days early compared with baseline eggs held outdoors but at the cost of a significantly lower overall hatch. There were no significant interactions between hatch location and species (F_4,45_ = 1.525, *p* = 0.220) or hatch location and storage treatment (F_4,45_ = 1.924, *p* = 0.113).

**Figure 2:**
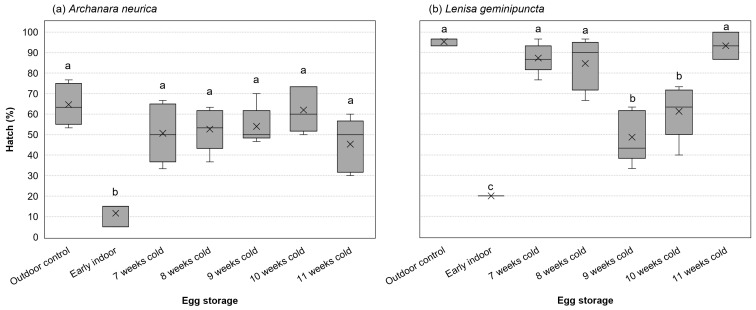
Effects of egg storage conditions on total egg hatch (% of initial eggs) of (**a**) *Archanara neurica* and (**b**) *Lenisa geminipuncta*. The early hatch treatment consisted of n = 3 dishes of 20 eggs for each of the species, and all other treatments consisted of n = 5 dishes of 30 eggs for each species. Group means are denoted by an “×”. Boxes depict the interquartile range with a horizontal bar for the median. Whiskers extend to the maximum and minimum data values. Letters denote Tukey groupings; within each species, means that do not share a letter are statistically significantly different at α = 0.05.

For eggs hatched in outdoor conditions, the percentage of eggs that successfully hatched varied with a significant interaction between species and storage (F_6,52_ = 8.104, *p* < 0.001, ω_p_^2^ = 0.43). Examining simple main effects by species, storage conditions affected outdoor hatch of both *A. neurica* (F_6,26_ = 8.55, *p* < 0.001, ω_p_^2^ = 0.58) and *L. geminipuncta* (F_6,26_ = 33.80, *p* < 0.001, ω_p_^2^ = 0.86). For *A. neurica*, eggs in the early hatch treatment experienced a 53% drop in hatch relative to baseline outdoor conditions, but otherwise, outdoor hatch was not affected by cold storage. For *L. geminipuncta*, eggs in the early hatch treatment experienced a 75% reduction in hatch relative to baseline outdoor conditions. The hatch of *L. geminipuncta* eggs stored outdoors after cold storage was comparable to the baseline outdoor treatment after 7, 8, and 11 weeks of cold storage and 34–47% lower after 9–10 weeks of cold storage (Figure 2).

**Figure 3:**
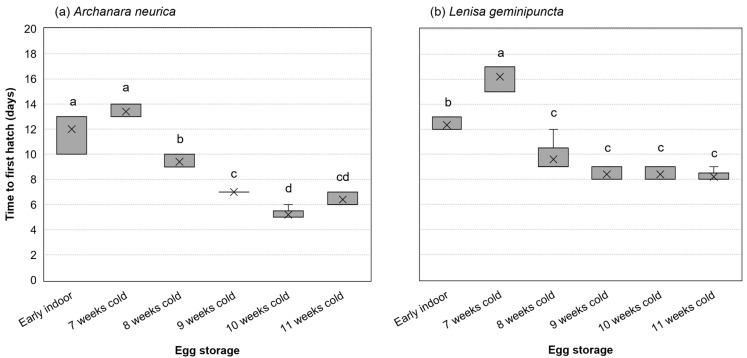
Time to first hatch (i.e., days between transfer to early indoor conditions or retrieval from cold storage to first hatch observation) of (**a**) *Archanara neurica* and (**b**) *Lenisa geminipuncta*. The early indoor treatment consisted of n = 3 dishes of 20 eggs for each of the species, and all other treatments consisted of n = 5 dishes of 30 eggs for each species. Group means are denoted by an “×”. Boxes depict the interquartile range with a horizontal bar for the median. Whiskers extend to the maximum and minimum data values. Letters denote Tukey groupings; within each species, means that do not share a letter are statistically significantly different at α = 0.05.

Time to first hatch following retrieval from cold storage was affected by an interaction of species and storage (F_5,44_ = 5.80, *p* < 0.001, ω_p_^2^ = 0.30). Examining simple main effects by species, storage conditions affected time to first hatch of both *A. neurica* (F_5,22_ = 104.8, *p* < 0.001, ω_p_^2^ = 0.95) and *L. geminipuncta* (F_5,22_ = 68.44, *p* < 0.001, ω_p_^2^ = 0.92). Time to first hatch of *A. neurica* was the longest for eggs brought indoors early or with the shortest cold storage of 7 weeks (12.0–13.4 days). As the duration of cold storage increased, time to first hatch generally became shorter by approximately four to eight days. Time to first hatch of *L. geminipuncta* was longest for eggs held in the cold for 7 weeks (16.2 days), intermediate for eggs brought indoors early (12.3 days), and shortest for eggs held in the cold for 8 to 11 weeks (Figure 3).

### 3.2. Monitoring Visual Signs of Egg Development to Predict Hatch Timing

**Figure 4:**
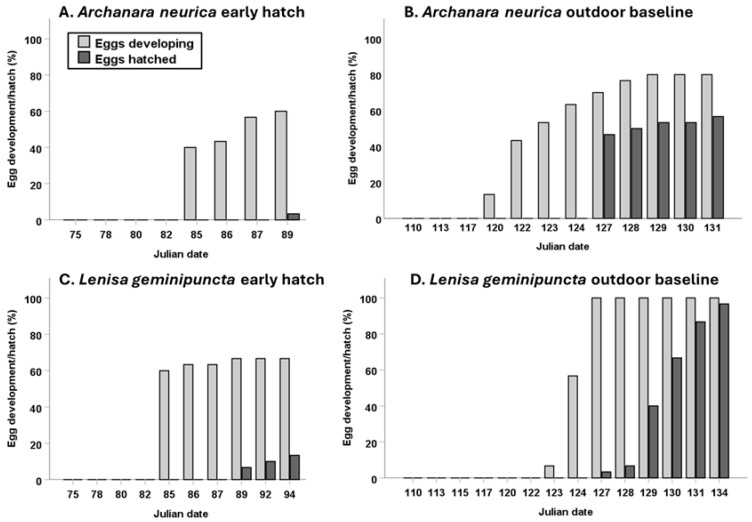
Egg development of *Archanara neurica* and *Lenisa geminipuncta* stored and hatched under outdoor baseline or early hatch conditions (one dish of 30 eggs per chart) over time (Julian date). Light gray bars indicate the cumulative percentage of eggs per treatment that have shown visible signs of development, and the dark gray bars depict the percentage of eggs per treatment that have hatched.

While formal statistical analyses were not possible on this low number of samples, the timing between visually detectable embryonation and hatch and the overall hatch duration appeared similar across species and storage/hatch conditions (Figure 4). The time from first signs of development to first hatch ranged from 4 to 7 days, and the length of the hatching period (hatch duration) was 1 to 8 days. A 1-day hatch duration occurred for the indoor *A. neurica* group, which had the lowest overall hatch (5%). Excluding this, the duration of the hatching period ranged from 5 to 8 days.

**Figure 5:**
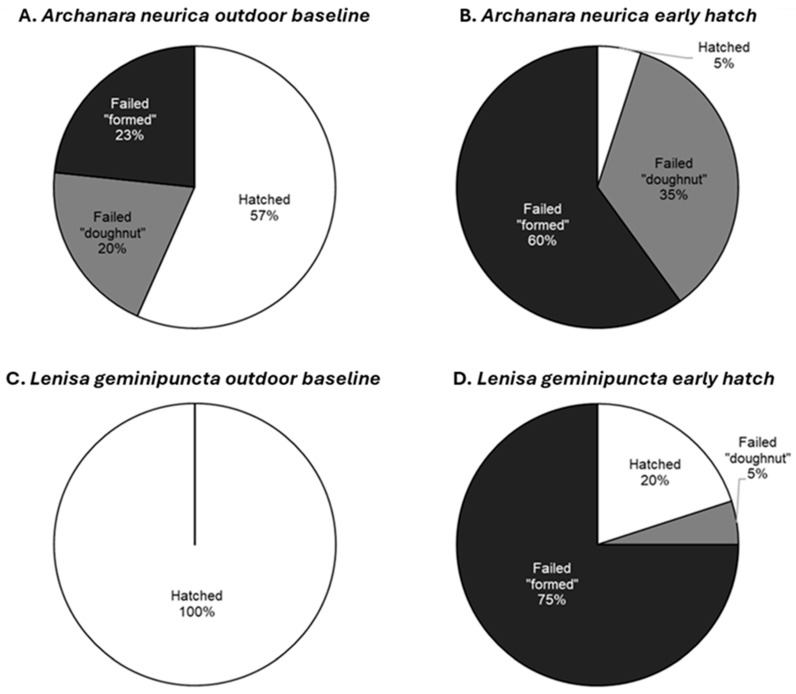
Final status of *Archanara neurica* and *Lenisa geminipuncta* stored and hatched under outdoor or indoor conditions (one dish of 30 eggs per chart). Photographic monitoring was used to classify eggs at the end of the study as hatched or failed in a characteristic “doughnut” or “formed” state (shown as % of eggs per dish allocated to each category).

For *L. geminipuncta*, 100% of eggs hatched successfully under baseline outdoor conditions. Only 5% of failed *L. geminipuncta* eggs took on the “doughnut” form, while this represented 20% and 35% of the failed *A. neurica* eggs in the outdoor baseline and early hatch treatments, respectively. For *L. geminipuncta* eggs in the early hatch treatment, 75% of eggs were “formed” but failed. For *A. neurica* eggs, the relative number of “formed” but failed eggs compared to the “doughnut” form was 23% vs. 20% in the outdoor baseline treatment and 60% vs. 35% in the early hatch treatment (Figure 5).

## 4. Discussion

The rate of successful egg hatch was significantly reduced for both *A. neurica* and *L. geminipuncta* when eggs were brought into the laboratory early in spring. For insects in temperate climates, overwintering eggs often need to be exposed to cold conditions for a threshold period of time in order to complete diapause and commence post-diapause development [35,36]. Indeed, the overwintering eggs of some polyphagous moth species have been shown to exhibit phenological polymorphisms, whereby populations have different cold duration requirements to synchronize the timing of spring hatch with the phenology of different host plants [37]. The low hatch rate of *A. neurica* and *L. geminipuncta* eggs brought into the laboratory early could, therefore, be explained by an insufficient duration of cold exposure. Cold exposure requirements of these insects may represent an adaptation to promote phenological synchronization with the spring emergence of *Phragmites*. Newly hatched larvae of *A. neurica* and *L. geminipuncta* need access to young *Phragmites* stems, and so the optimal hatching window in the field is relatively narrow [27]. It should be noted, however, that all *A. neurica* and *L. geminipuncta* eggs that were hatched in indoor conditions, even those that had experienced extended cold exposures, exhibited very low hatch rates. An alternative explanation may be that egg hatch in *A. neurica* and *L. geminipuncta* is negatively affected by a lack of temperature fluctuation, as has been observed in other Noctuid moths [38].

For *A. neurica* eggs hatched outdoors, the percentage of eggs that successfully hatched was not affected by any of the cold storage treatments. In the longest cold storage treatment, egg hatch was delayed by up to 31 days, compared with outdoor controls, without any negative impact on viability. This is an important finding for the biological control program, as it will allow practitioners to dramatically extend the time during which larval releases, the most effective release method to date [3,39], can be conducted.

For *L. geminipuncta* eggs hatched outdoors, egg hatch percentage was not affected by cold storage for 7, 8, or 11 weeks; however, a significant reduction in egg hatch was observed after 9 and 10 weeks of cold storage. This dip in hatch rate is difficult to explain, as it did not coincide with any large fluctuations in storage temperatures. The reprise of high hatch rates after 11 weeks of cold storage, however, suggests that this dip was associated with post-cold storage conditions, or the transition to outdoor conditions, and not cold storage itself. Both relative humidity [40,41] and rapid temperature fluctuations [42], for example, can impact post-diapause egg development in insects and should be the focus of future work.

Time to first hatch was measured for each cold treatment, starting from the date that the eggs were moved out of cold storage and into outdoor conditions. For both *A. neurica* and *L. geminipuncta*, time to first hatch became shorter as the duration of cold storage increased. Given that the rate of accumulation of degree days, after removal from cold storage, was similar among treatments, these results suggest that both *A. neurica* and *L. geminipuncta* were able to undergo some cumulative development while in cold storage conditions. Minimum temperature thresholds for egg development vary widely among Noctuid moths [43,44]. Our results suggest a threshold temperature below 5 °C for eggs of *A. neurica* and *L. geminipuncta*.

We also conducted a visual analysis of *A. neurica* and *L. geminipuncta* eggs to see if the timing of hatch could be predicted from changes in the appearance of eggs. For both *A. neurica* and *L. geminipuncta*, egg hatch occurred 4–7 days after the first visible signs of egg development, and hatch duration (time from first to last hatch) generally ranged from 5 to 8 days. We do not want to lean too heavily on this relatively small pilot study, and future work should seek to verify these results. Once verified, these results will provide practitioners with important cues for predicting peaks of agent hatch. In order for larval releases to be conducted, newly emerging larvae must be placed inside cut *Phragmites* stems within c. 24 h of hatching. Larvae left outside of stems for longer than this usually desiccate and die [3]. Waves of agent hatch, therefore, require the prior collection of large numbers of *Phragmites* stems, which can only be stored for approximately one week, as well as the immediate attention of trained practitioners. Predicting peaks and troughs of agent hatch will allow for more efficient use of larvae, as well as practitioner time and resources.

The percentage of eggs that failed to hatch and exhibited the doughnut appearance was 20% and 35% for *A. neurica* for the early hatch and the outdoor baseline treatments, respectively. For *L. geminipuncta*, failed eggs with the doughnut appearance represented 0% and 5% of early hatch and outdoor baseline eggs, respectively. These results suggest a higher proportion of non-viable eggs in *A. neurica* than *L. geminipuncta*, which is in line with consistent observations from the mass rearing programs in Switzerland (Patrick Hafliger, personal communication) and what we have observed in Canada. For the early hatch group, eggs were moved to indoor conditions early in the spring, and many eggs that failed to hatch showed visible signs of development (60% of *A. neurica* and 75% of *L. geminipuncta* eggs). This suggests that the shortened exposure to natural cold conditions did not prevent eggs from completing diapause and that indoor conditions affected some elements of post-diapause development. Indeed, previous studies have suggested that, in southern Europe, *L. geminipuncta* diapause is completed in early winter [45].

Using cold storage to manipulate the timing of egg hatch in *A. neurica* and *L. geminipuncta* will allow biological control practitioners to substantially increase the number of larvae available for releases during the spring, as well as optimize the timing of releases. We recommend that eggs be placed in cold storage in early spring, prior to any unseasonably warm weather that might stimulate early development. Eggs should then be removed from cold storage on a timescale that maximizes their efficiency of use. Eggs should be removed from cold storage a maximum of four weeks after the mean hatch date of outdoor control eggs, and all eggs should be held in outdoor conditions for hatching.

## Figures and Tables

**Table 1:** Temperature data from egg storage and hatching locations. The experiment started on 15 March 2024. The cold storage refrigerator was used until 29 May 2024, and hatch monitoring occurred outdoors in the courtyard shed and indoors on a laboratory bench until 13 June 2024.

Location	Date Range	Mean Temperature ± SD (°C)	Min Temperature (°C)	Max Temperature (°C)	Temperature Range (°C)	Coefficient of Variation (CV)
Outdoor hatch (courtyard shed)	15 March– 13 June	12.1 ± 6.3	−4.3	25.8	30.1	0.52
Cold storage (refrigerator)	15 March– 29 May	4.6 ± 1.5	3.6	27.3	23.7	0.32
Early hatch (laboratory bench)	15 March– 13 June	23.4 ± 2.1	16.7	27.7	11	0.09

**Table 2:** Cumulative degree days at Tbase = 5 °C and Tbase = 0 °C from the start of the cold storage experiment on 15 March 2024 until the date of mean first hatch of *A. neurica* and *L. geminipuncta* combined for each storage condition.

Storage Conditions	Cumulative Degree Days (Tbase = 5 °C)	Cumulative Degree Days (Tbase = 0 °C)
Outdoor baseline	201	418
Early hatch	218	278
7 weeks cold	168	440
8 weeks cold	134	414
9 weeks cold	132	432
10 weeks cold	112	437
11 weeks cold	120	480

## Data Availability

The data that support the findings of this study are available at https://osf.io/7ycxs/files/xnmd4 (accessed on 20 January 2026).
